# A global dataset on tools, frameworks, and indicator sets for smart city assessment

**DOI:** 10.1016/j.dib.2020.105364

**Published:** 2020-03-02

**Authors:** Ayyoob Sharifi

**Affiliations:** a Graduate School for International Development and Cooperation, Hiroshima University, Higashi-Hiroshima 739-8530, Japan; b Network for Education and Research on Peace and Sustainability (NERPS), Japan

**Keywords:** Smart city indicators, Dataset, Assessment tools, Assessment methods, Typology, Measurement, Evaluation, Urban planning

## Abstract

This is a dataset on the typology, content, and structure of 34 selected schemes (i.e., tools, frameworks, indices, indicator sets) for assessing city smartness. The data is collected through content analyses of related documents such as manuals, working papers, and scorecards. The dataset covers aspects related to development as well as implementation of assessment schemes. It provides details related to the geographic and thematic scope of each assessment scheme, approaches towards indicator selection, themes that are consistently used across the schemes, indicators used for smart city assessment, methods applied for performing assessment, the state of implementation, and the complete list of cities that have been evaluated and their rankings (if applicable). The dataset is related to the research article titled “A typology of smart city assessment tools and indicator sets”.

**Table:** Specifications Table

Subject area	Geography, Planning and Development
Specific subject area	Smart Cities
Type of data	Table, graph, figure
How data were acquired	Compiled through content analyses of 34 selected smart city assessment schemes. For a completed list of the selected schemes see Sharifi [1]
Data format	Raw, analyzed
Parameters for data collection	Indicators used for smart city assessment, development trends, typology of the tools, thematic focus, method of development, format of the schemes, scoring and weighting approaches, implementation status
Description of data collection	A literature survey was conducted to select relevant assessment schemes. Next, matrices were developed in Microsoft Excel to collect data related to various aspects related to content and structure of the selected schemes. Matrix rows and columns corresponded to selected schemes and the explored aspects, respectively. The matrix was completed through content analyses of the schemes. Wherever necessary, simple descriptive statistical analyses using Microsoft Excel
Data source location	Institution: Various institutions from across the world. Region: Global The full list of institutions and regions is available in the manuals, working papers, and web pages related to the 34 selected assessment schemes. For details see Table 1 of the related research article.
Data accessibility	Data are mainly within this article. Data related to the indicators and assessed cities/city rankings are available as supplementary appendices.
Related research article	A. Sharifi, A typology of smart city assessment tools and indicator sets, Sustainable Cities and Society, (2019) 101936. https://doi.org/10.1016/j.scs.2019.101936 [1]

**Table:** 

**Value of the Data** • This dataset is the first of its kind to provide details on the content and structure of smart city assessment schemes. • The dataset can serve as a frame of reference for those researchers wishing to further analyze smart city assessment schemes. • Scheme developers can use the dataset to become familiar with other existing tools and to explore opportunities for knowledge exchange and mutual learning. • End-users (e.g., city authorities, investors, etc.) can use the dataset to choose the assessment scheme that best fits their needs and priorities.

## 1 Data

**Fig. 1:**
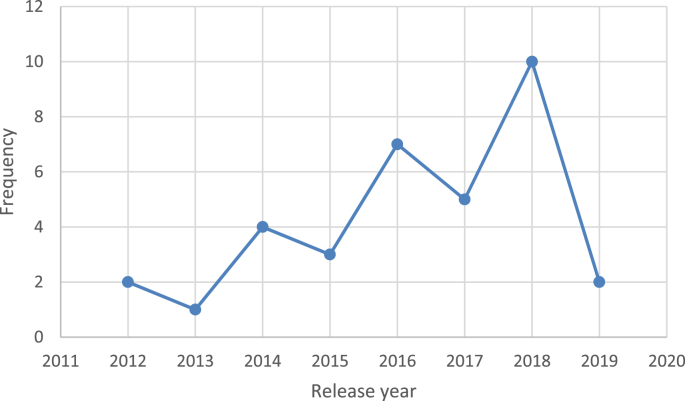
The year of development of the selected schemes.

**Fig. 2:**
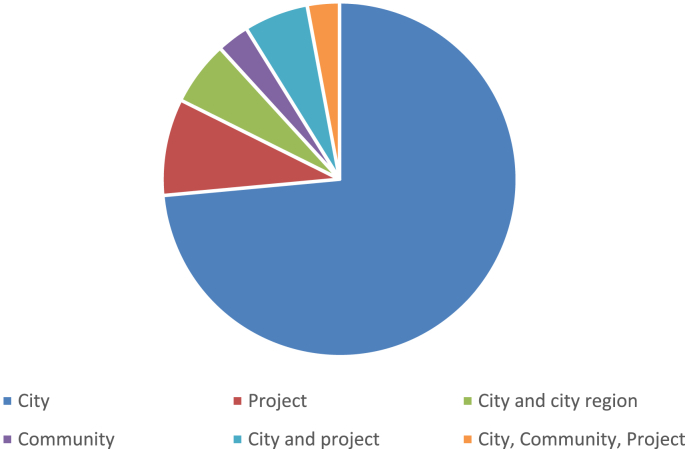
Frequency distribution of the schemes based on their scale of analysis.

**Fig. 3:**
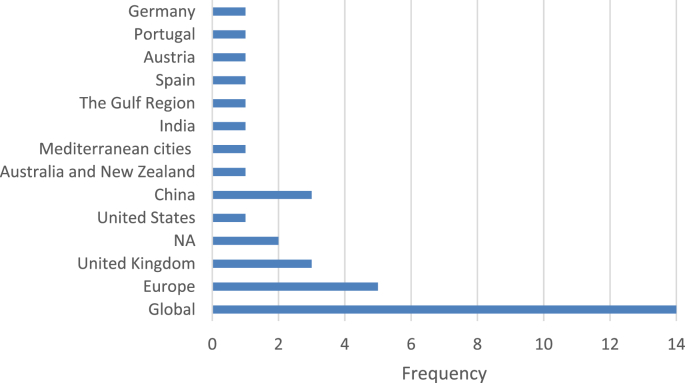
Geographic focus of the selected assessment schemes.

**Fig. 4:**
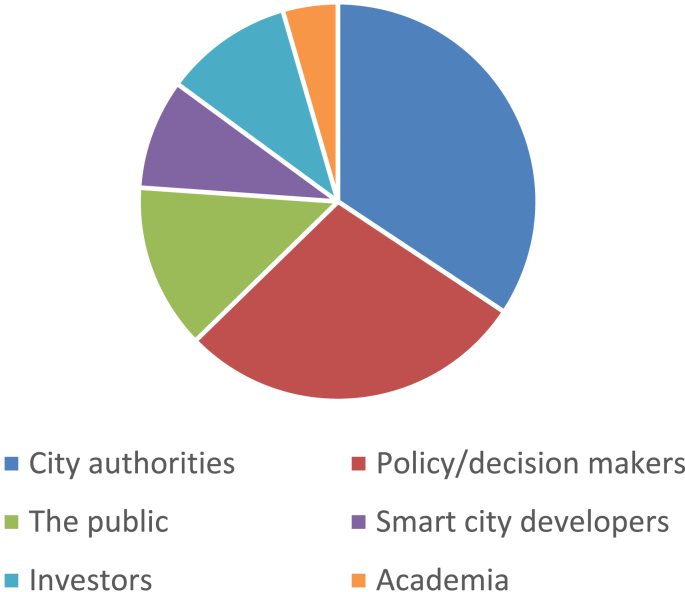
The target audiences of the selected schemes.

**Fig. 5:**
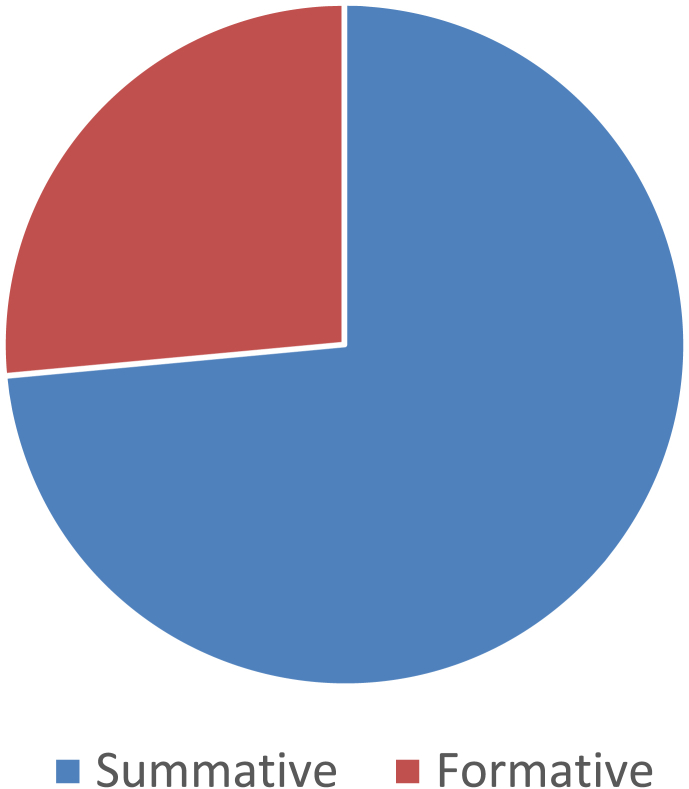
Major approaches towards assessment.

**Fig. 6:**
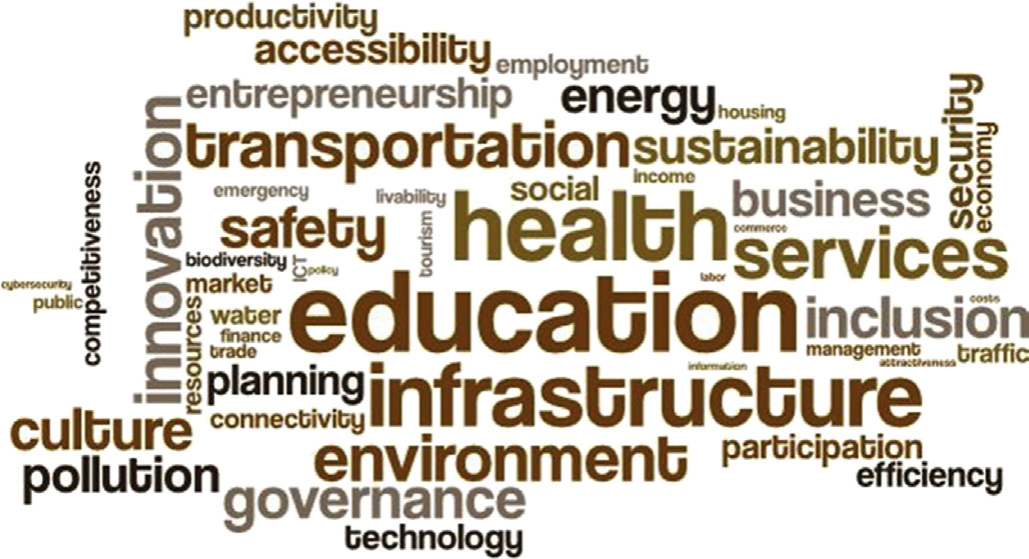
Word-cloud visualization of the persistently-used factors across the schemes.

**Fig. 7:**
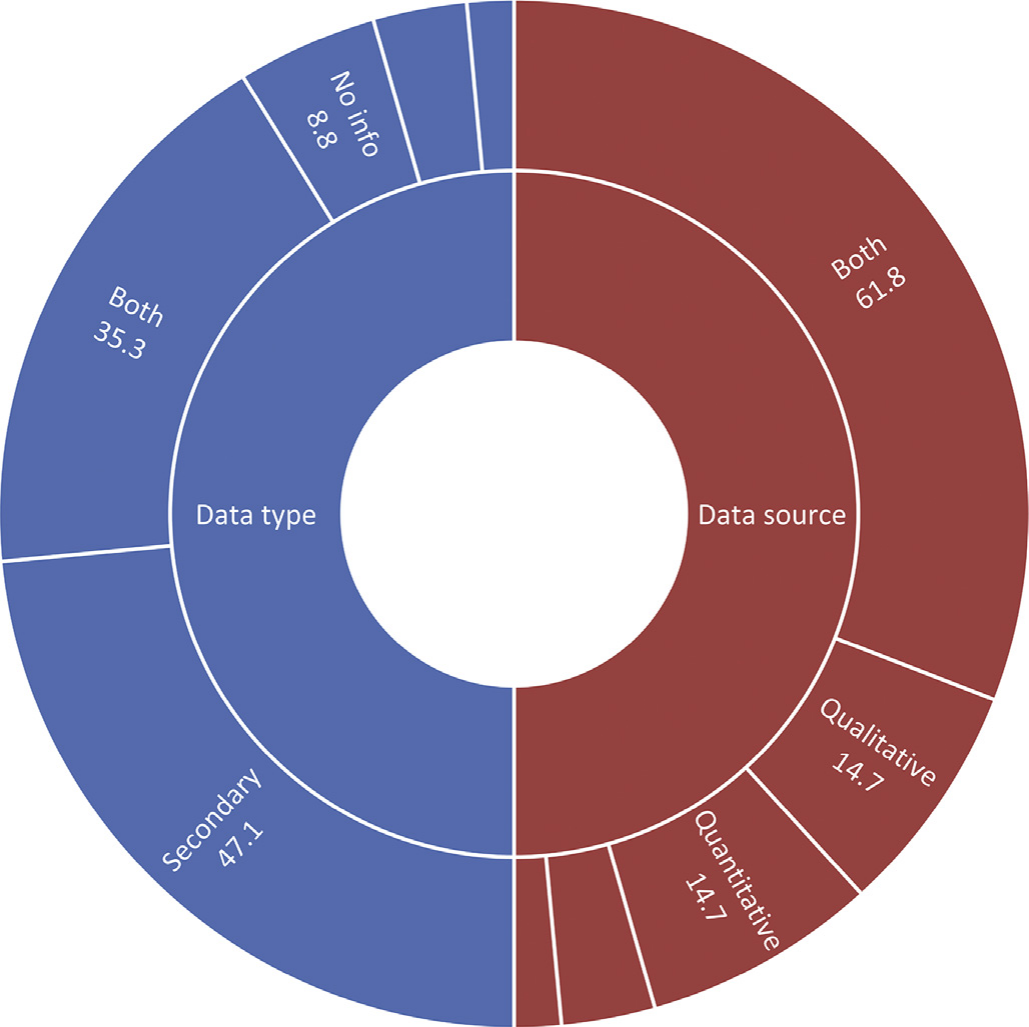
Percentage distribution of the selected schemes according to data type and source.

**Fig. 8:**
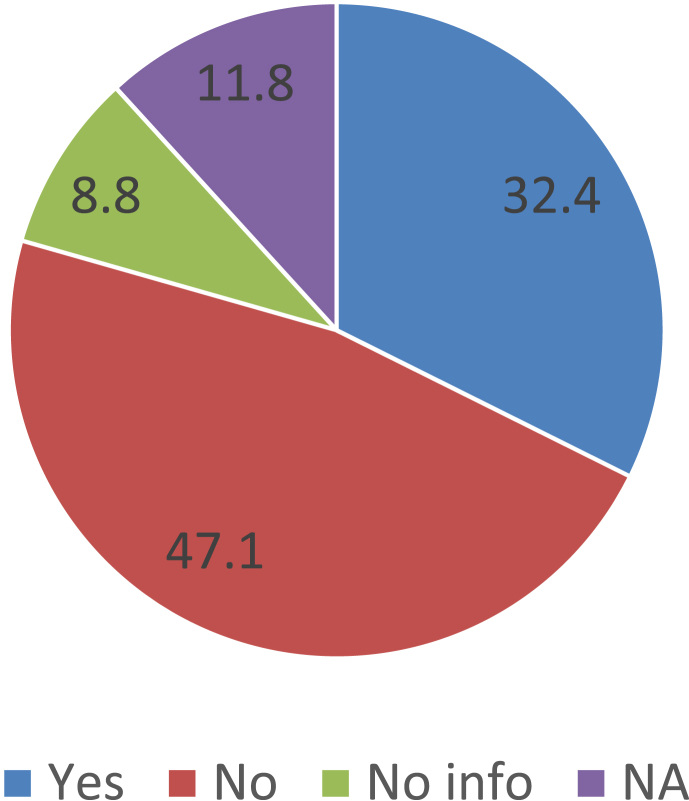
Percentage distribution according to whether or not the schemes have adopted equal weighting.

**Fig. 9:**
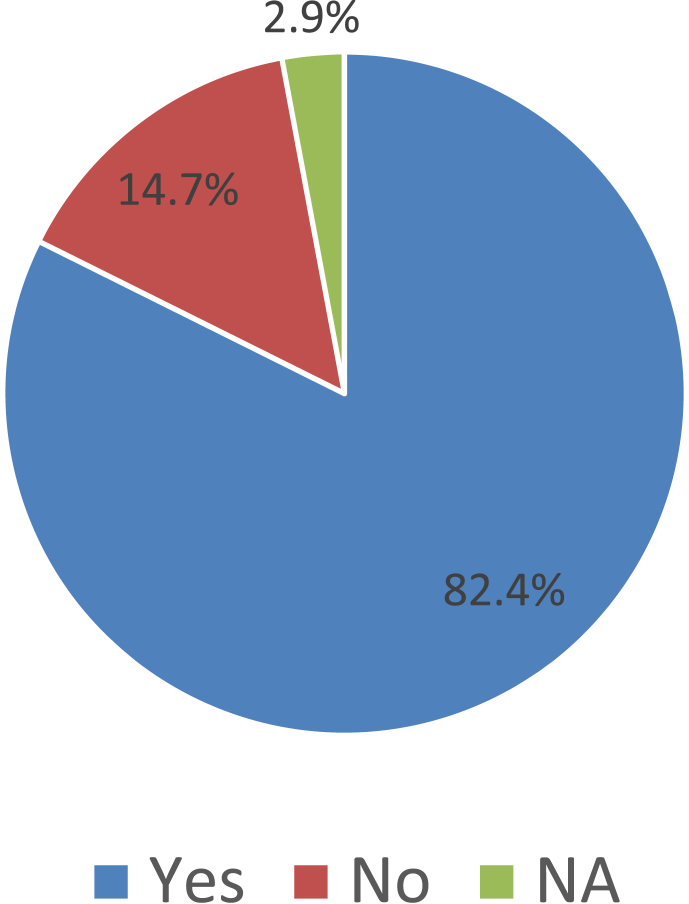
The extent of application of the selected schemes.

**Fig. 10:**
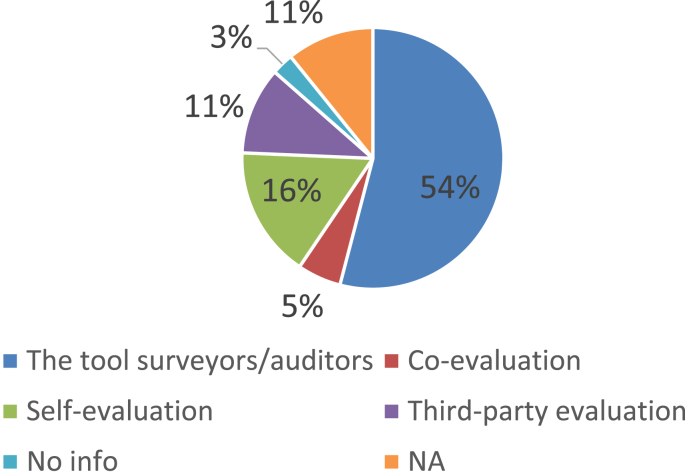
Distribution percentage according to the assessment body.

Over the past ten years or so, many ‘tools, frameworks, and indicator sets’ (hereafter, ‘schemes’) have been developed for smart city assessment [2]. Data related to the content and structure of 34 selected schemes is collected and presented here (see Table 1 of the related research article [1] for the complete list of the selected schemes). The dataset is not intended to be exhaustive or a representative sample of all existing schemes. However, efforts have been made to include all relevant schemes published until December 2018. Table1 presents data related to the general characteristics of the selected assessment schemes. Fig. 1 shows the number of schemes developed in each year. In Fig. 2 frequency distribution of the schemes based on their scale of analysis is presented. Fig. 3 displays geographic focus of the selected assessment schemes. Fig. 4 shows the major target audiences of the selected schemes. Fig. 5 displays major approaches towards assessment. Table 2 presents thematic focus and main themes of each assessment scheme. In Fig. 6 word-cloud visualization of the persistently-used factors across the schemes is shown. Table 3 displays data on the format of each of the assessment schemes. Table 4 presents different types of labels used by the assessment schemes. Scheme-by-scheme information on data types/sources and weighting approaches is presented in Table 5. Fig. 7. Displays percentage distribution of the selected schemes according to data type and source. Fig. 8 presents percentage distribution according to whether or not the schemes have adopted equal weighting. Table 6 provides data on the approaches towards scoring. Table 7 presents data on assessment bodies and the extent of implementation. Fig. 9 displays the extent of application of the selected schemes. Fig. 10 shows which entities have conducted the assessment process. Appendix I provides the list of compiled indicators. Appendix II provides data related to the cities evaluated using at least one of the selected assessment schemes, along with their rankings (wherever applicable).

**Table 1:** General characteristics of the selected assessment schemes.

Scheme	Scale	Geographic focus	Method of development	Main target audience	Formative/summative
LRSC	City	European Union	Literature review	Policy/decision makers	Summative
SSCC	City	Chinese cities	Literature review, expert opinion	Policy/decision makers	Summative
CIMI	City	Globally representative	Literature review	The public (citizens) and local governments	Summative
GPCI	City	Globally representative	Researcher and expert opinions	City authorities, policy/decision makers	Summative
ICI	city (also states and regions)	Globally representative	Expert opinions (advice)	City authorities	Summative
EP	City	Globally representative	Expert opinions	The public, policy/decision makers (transport policy)	Summative
IES-City	City Projects	Globally representative (generic)	Literature review, expert opinion	City authorities, policy/decision makers and regulators, vendors/integrators, standard development organizations, researchers	Formative
SCC	City	Globally representative (generic)	Expert opinions	Built environment consultants, city authorities	Formative
WWC	City	United States	Expert opinions	City authorities, local governments	Summative
CSC	Community (precinct, neighborhood, town centers, campuses)	Australia and New Zealand	Expert opinions	State and local governments, private development sector	Formative
CSCP	City	Chinese cities	Literature review, expert opinion	city officials, planners and decision makers	Summative
SCG	City	Globally representative	Expert opinions	City authorities	Summative
ASCIMER	City projects	Mediterranean cities	Literature review, expert opinion, lessons from best practices of assessment	City authorities, policy/decision makers, public/private investors	Formative
SCI-I	City	Indian cities	Literature review, expert opinions, stakeholder consultation workshops	City government, state and central government, the public, investors	Summative
U4SSC	City	Globally representative	Experts from the main developers, in consultation with relevant stakeholders and member States	City authorities, other parties such as the UNECE (United Nations Economic Commission for Europe)	Formative
Juniper	City	Globally representative	Expert opinions	Citizens, policy/decision makers	Summative
UK-SCI	City	UK cities	Literature review, expert opinion	Policy/decision makers, businesses and industry stakeholders	Summative
CITYkeys	City and project scales	European cities	Literature review, expert consultation workshops	City authorities, policy/decision makers, business and industrial stakeholders, smart city developers and service providers, the public, education and knowledge institutes	Summative
NSCI	City	Globally representative	Literature review, expert opinions, best practice analysis	Policy/decision makers	Summative
CoO	City	Globally representative	Literature review, available indices	The public, city authorities, investors	Summative
CKPI	City, neighborhood, project	Manchester (the UK)	Co-designed with stakeholders and community members	Policy/decision makers, city authorities, the public	Formative (can be highlighted as the best example of formative tool)
GSSCI	City	The Gulf Region	Literature review, expert opinions, public survey	Policy/decision makers	Summative
EDCi	City	European cities	Literature review, expert opinions	Policy/decision makers, entrepreneur, and researchers	Summative
SCSGM	City	Globally representative (generic)	Literature review, expert opinions, public survey	City authorities	Summative
City-IQ	City	Globally representative	Literature review, expert opinions	City authorities, policy/decision makers, smart city developers and service providers	Formative
IDC	City	Spain, Portugal, and Germany	Expert opinions	City authorities, smart city developers and service providers	Formative
ITU-T	City, city region	Globally representative (generic)	Focus group discussions, literature review	City authorities, policy/decision makers, the public, smart city developers and service providers, evaluation agencies and the academia	Summative
EU-MSC	City	Medium-size European Cities	Consultation workshops	Policy/decision makers, investors	Summative
Boyd-Cohen	City	Globally representative	Expert opinions	City authorities, policy/decision makers	Summative
MSC-EU	City projects	EU cities	Literature review, expert opinions	Policy/decision makers, investors	Summative
SCMM	city, city projects	Scottish cities	Based on the IDC maturity model	City authorities	Formative
SCP	City	Austrian Cities	Expert-based in collaboration with participating cities (workshops and feedback)	City authorities, climate change mitigation and adaptation policy making	Summative
UCLG	City	Globally representative	Expert opinions	City authorities	Summative
SCBC	City	Chinese cities	Expert opinions	Smart city infrastructure providers	Summative

**Table 2:** Thematic focus and main themes of each assessment scheme.

Scheme	Emphasis	No. of themes	No. of factors	No. of indicators	Main themes
**LRSC**	Economy, environment, culture and society	3		32	Economy, Environment, Society and Culture
**SSCC**	economy, IT, mobility	6	16	22	Technological innovation, smart economy, smart infrastructure, smart services, smart mobility, smart environment
CIMI	General smartness and sustainability	9	–	83	Human capital, social cohesion, economy, governance, environment, mobility and transportation, urban planning, international outreach, and technology
GPCI	Competitiveness	6	26	70	Economy, research and development, cultural Interaction, livability, environment, and accessibility"
ICI	Innovative economy	3	31	162	Cultural assets, human infrastructure, networked markets
EP	Mobility	8	24	39	transport and mobility, sustainability, governance, innovation economy, digitalization, cyber Security, living standard, expert perception
IES-City	Readiness, interoperability, scalability, and investment priority identification	8	–	128	Economy, environment, society, strategic intent, data, ICT infra and technologies, governance and service delivery models, stakeholder engagement
SCC	Livability, workability, and sustainability	8	–	31 (43 if supplements are considered)	Built environment, energy, telecommunications, transportation, water and wastewater, health and human service, public safety, payments and finance
WWC	Data-driven governance	8	–	45	Data Governance, evaluations, general management, open data, performance & analytics, repurposing, results-driven contracting, stakeholder engagement
CSC	Sustainability, livability, and productivity	5	–	32	Strategicness, connectivity, awareness, responsiveness, innovation
CSCP	General smartness	5		18	Infrastructure, people, governance, economy, and environment
SCG	Governance		10		Vision, leadership, budget, financial incentives, support programs, talent-readiness, people-centricity, innovation ecosystem, smart policies, track record
ASCIMER	Efficiency, sustainability, resilience, quality of life (QOL), and finance-ability	6	36	133	Mobility, economy, governance, living, people, environment
SCI-I	General smartness	6	34	58	Living, governance, people, economy, mobility, environment
**U4SSC**	Focus on SDGs and sustainability in addition to smartness	3	22	91	Economy, environment, and society and culture
Juniper	Saving benefits of smart solutions related to mobility, healthcare, public safety, productivity	4	–	75	Mobility, healthcare, public safety, productivity
UK-SCI	ICT technology, readiness, and maturity	2	10	–	Strategy, execution
CITYkeys	General smartness	5 (project level) 4(city)	22	101 (project)76 (city)	People, Planet (environment), prosperity (economy), governance, propagation (only for project level)
NSCI	ICT maturity and sustainability	6	16	37	ICT Maturity (infrastructure, affordability, use); TBL sustainability (social, economy, environment)
CoO	Innovative economy and QOL	3	10	67	Tools for a changing world, QOL, economics
CKPI	User acceptance (perception) of technologies and services	–	–	–	Should be defined by stakeholders in a participatory process
GSSCI	Future smartness strategies (and execution)	2	–	10	Smart city strategy and execution
EDCi	Digital Entrepreneurship		10	40	Access to capital, business environment, digital infrastructure, entrepreneurial culture, knowledge spillovers, lifestyle, market, mentoring and managerial assistance, non-digital infrastructure, skills
SCSGM	Smart city strategy and organisational mechanisms of a smart city	10	–	100	City strategy, stakeholder engagement & communication, operating model & service delivery, physical asset management, data strategy, access to data, ICT plan, standards, innovation ecosystem, performance management
City-IQ	General smartness	5	–	20	environment & construction, governance & public service, economy & industries, informatization, innovative human resource
IDC	General smartness	8	23 (22 for Germany)	58 (47 for Portugal, 65 for Germany)	Enabling factors (people, economy, iCT); Dimensions (government, buildings, mobility, energy and environment, services)
ITU-T	Smartness and sustainability	6	28	90	ICT, environmental sustainability, productivity, quality of life, equity and social inclusion, physical infrastructure
EU-MSC	General smartness and QOL	6	28	81	Economy, people, governance, mobility, environment, living
Boyd-Cohen	General smartness	6	18	62	Economy, government, people, living, mobility, environment
MSC-EU	General smartness	6	not mentioned	not mentioned	Governance, people, living, smart mobility, economy, environment
SCMM	Smart city maturity	5	Will be selected locally	Will be selected locally	Strategic Intent, data, technology, governance and service delivery models, stakeholder engagement (cross-cutting capabilities across different domains)
SCP	Climate and Energy	5	–	21	Buildings & settlement structures, transport & mobility, technical infrastructure, economy & population and policy, administration & governance
UCLG	General smartness	6	22	48	Economy, people, governance, mobility, environment, living
SCBC	Smart Infrastructure for instrumentation	5	19	64	Infrastructure, public management and service, information service for economic development, culture and science, sense of citizen

**Table 3:** Format of the assessment schemes.

Scheme	Format	Composite index	Ranking	Labelling
**LRSC**	Index	Yes	Yes	No
**SSCC**	Index	Yes	Yes	Yes
CIMI	Index	Yes	Yes	Yes
GPCI	Index	Yes	Yes	No
ICI	Index	Yes	Yes	Yes
EP	Index	Yes	Yes	No
IES-City	Toolkit (no index)	NA	No	NA
SCC	Scorecard	No	No	NA
WWC	Scorecard	Yes	Yes	Yes
CSC	Toolkit	No	No	NA
CSCP	Index	Yes	Yes	Yes
SCG	index	Yes	Yes	No
ASCIMER	Toolkit	Yes	Yes	Yes
SCI-I	Index	Yes	Yes	No
**U4SSC**	Toolkit	Yes (planned)	No	No
Juniper	Index	Yes	Yes	No
UK-SCI	Index	Yes	Yes	Yes
CITYkeys	Scorecard	No	No	No
NSCI	Index	Yes	Yes	No
CoO	Index	Yes	Yes	Yes
CKPI	Toolkit	No	No	NA
GSSCI	Index	Yes	Yes	Yes
EDCi	Index	Yes	Yes	No
SCSGM	Scorecard	No	No	Yes
City-IQ	Index	Yes	Yes	No
IDC	Index	Yes	Yes	yes
ITU-T	Toolkit (no index)	NA	NA	NA
EU-MSC	Index	Yes	Yes	No
Boyd-Cohen	Index	Yes	Yes	Yes
MSC-EU	Index	No	Yes	No
SCMM	Toolkit	No	No	NA
SCP	Index	No	No	No
UCLG	Scorecard	No	No	NA
SCBC	Index	Yes	Yes	Yes

**Table 4:** Different types of labels used by the assessment schemes.

Scheme	Label
SSCC	The first group, the second group, the third group, the fourth group, the fifth group
CIMI	A (90–100), RA (60–90), M (45–60), and B (below 45)
ICI	Nexus, Hub Cities, Node Cities, Upstart, Unclassified
WWC	Platinum, Gold, Silver
CSCP	Best, good, average, poor, and worst
ASCIMER	Very poor, poor, acceptable, good, very good
UK-SCI	Leaders, contenders, challengers, followers
CoO	High, Medium, Low
GSSCI	Leaders (above 75), Contenders (above 50), Challengers (above 25), and Followers
SCSGM	maturity levels: Ad hoc, opportunistic, repeatable, managed, optimized
IDC	Top five, contenders, players, followers
Boyd-Cohen	Pioneering, emerging, next Stage
SCMM	Maturity levels: ad-hoc, opportunistic, Purposeful & Repeatable, operationalized, optimized
SCBC	Leader, follower, preparing

**Table 5:** Scheme-by-scheme information on data type/source and weighting approaches.

Scheme	Data type	Data source	Equal weighting
LRSC	Secondary	Both	No
SSCC	Secondary	Quantitative	No
CIMI	Secondary	Both	No
GPCI	Secondary	Quantitative	Yes
ICI	Secondary	Both	No info
EP	Secondary	Both	No
IES-City	No info	Qualitative	NA
SCC	Both	Qualitative	Yes
WWC	Primary	Qualitative	Yes
CSC	Both	Both	Yes
CSCP	Secondary	Both	No
SCG	No info	No info	yes
ASCIMER	Both	Both	No
SCI-I	Both	Both	No
U4SSC	Secondary	Both	No info
Juniper	Secondary	Both	No
UK-SCI	Both	**Both**	No
CITYkeys	Both	Both	Yes
NSCI	Secondary	Both	No
CoO	Secondary	Both	Yes
CKPI	No info	No info	NA
GSSCI	Both	Both	No
EDCi	Secondary	Both	No
SCSGM	Primary	Qualitative	Yes
City-IQ	Secondary	Both	Yes
IDC	Both	Both	No
ITU-T	NA	NA	NA
EU-MSC	Secondary	Both	No
Boyd-Cohen	Both	Both	Yes
MSC-EU	Secondary	Quantitative	No
SCMM	Both	Qualitative	No info
SCP	Secondary	Quantitative	Yes
UCLG	Both	Both	NA
SCBC	Both	Quantitative	no

**Table 6:** Data on the approaches towards scoring.

Scheme	Benchmarking	Baseline	Maturity model	Against peers	Targets	Scenario making
LRSC	Yes	No	No	Yes	No	No
SSCC	Yes	No	No	Yes	No	No
CIMI	Yes	No	No	Yes	No	No
GPCI	Yes	Yes	No	No	No	No
ICI	Yes	Yes	No	No	Yes	No
EP	Yes	Yes	No	Yes	No	No
IES-City	No	No	Yes	No	No	No
SCC	No	Yes	Yes	No	No	No
WWC	Yes	Yes	Partial	No	No	No
CSC	No	No	Yes	No	No	No
CSCP	Yes	Yes	No	Yes	No	No
SCG	Yes	Yes	Yes	No	No	No
ASCIMER	No	No	No	No	Yes	No
SCI-I	Yes	Yes	No	Yes	No	No
U4SSC	No	Yes	No	No info	Yes	No
Juniper	Yes	Yes	No	No	Yes	Yes
UK-SCI	Yes	Yes	Yes	No	Yes	No
CITYkeys	Yes	yes	Yes	No	No	No
NSCI	Yes	Yes	Partial	yes	No	No
CoO	Yes	No	No	Yes	Yes	Partial
CKPI	Yes	No	No	No	No	No
GSSCI	Yes	Yes	Yes	No	Yes	No
EDCi	Yes	Yes	No	Yes	No	No
SCSGM	No	No	yes	No	No	No
City-IQ	Yes	Yes	No	yes	No	Yes
IDC	Yes	Yes	Yes	No	No	No
ITU-T	NA	NA	Na	NA	NA	NA
EU-MSC	Yes	Yes	No	Yes	No	No
Boyd-Cohen	Yes	Yes	No	Yes	No	No
MSC-EU	Yes	No	Yes	No	Yes	No
SCMM	No	Yes	Yes	No	No	Yes
SCP	Yes	Yes	No	Yes	No	No
UCLG	Yes	No	No	No	Yes	No
SCBC	Yes	No	No	No	Yes	No

**Table 7:** Data on assessment bodies and the extent of implementation.

Scheme	Who evaluates	Applied	No. of cities evaluated	Rationale for selecting cities
LRSC	The tool surveyors/auditors	Yes	28	Based on size political position (“capital metro regions” of Europe)
SSCC	The tool surveyors/auditors	Yes	35	Based on political position and reputation for smart city activities
CIMI	The tool surveyors/auditors	Yes	165	Based on data availability and considering size, socio-economic, cultural, and political importance
GPCI	The tool surveyors/auditors	Yes	44	Three criteria: 1- top 10 cities in influential city ranking systems; 2- major cities of top 10 globally competitive cities; 3- other cities that are deemed appropriate for inclusion by the experts and tool developers
ICI	The tool surveyors/auditors	Yes	500	“Based on basic factors of health, wealth, population, geography as well as potential relative to peers"
EP	The tool surveyors/auditors	Yes	121	No info
IES-City	No info	No	NA	NA
SCC	The city analysts/auditors	No	NA	NA
WWC	Third-party surveyors/auditors	Yes	9	Population size
CSC	Self-evaluation	No	NA	NA
CSCP	The tool surveyors/auditors	Yes	44	Data availability and reputation for smart initiatives
SCG	The tool surveyors/auditors	Yes	50	Literature review on ranking and assessment practices
ASCIMER	European Investment Bank (EIB) evaluators	Yes	5	Data availability, existence of collaboration with experts (in the selected cities) who have collaborated to the development of the assessment methodology
SCI-I	Third part surveyors/auditors	Yes	53	Population size
U4SSC	The city analysts/auditors; Third-party surveyors/auditors	Yes	100	No info
Juniper	The tool surveyors/auditors	Yes	20	No info
UK-SCI	The tool surveyors/auditors	Yes	20	Reputation for smart city development
CITYkeys	Self-evaluation	Yes	50	Data availability
NSCI	The tool surveyors/auditors	Yes	40	Global coverage (geographic balance), reputation for strength in smart city development
CoO	The tool surveyors/auditors	Yes	30	Being a capital market center, global coverage (geographic balance)
CKPI	Co-evaluation with stakeholders	Pilot-test	1	NA
GSSCI	The tool surveyors/auditors	Yes	10	Reputation, leading cities in the Gulf region in the area of ICT
EDCi	The tool surveyors/auditors	Yes	60	“All capital cities in the EU28”. “Additionally, it includes 32 non-capital cities in the EU that are important hubs of digital entrepreneurship"
SCSGM	Either self-evaluation or third-party evaluation	No	NA	NA
City-IQ	The tool surveyors/auditors	Yes	41	Reputation; eight highly ranked Chinese cities and 33 European and the US cities that had promoted iCity construction concepts worldwide and that had implemented iCity construction practices for a long time".
IDC	The tool surveyors/auditors	Yes	146	Population size
ITU-T	NA	NA	NA	NA
EU-MSC	The tool surveyors/auditors	Yes	77	Population size, data availability, location in the region
Boyd-Cohen	Self-evaluation	Yes	100	Global coverage (geographic balance), data availability; for 2014 version selected based on their Innovation Cities Index Score (reputation)
MSC-EU	The tool surveyors/auditors	Yes	20	data availability, population size, geographical location; status of the city (reputation), etc.
SCMM	City-nominated experts in collaboration with stakeholders (co-evaluation)	No	NA	NA
SCP	The tool surveyors/auditors	Yes	12	Population size, reputation for interest and activities related to smart solutions for climate and energy planning
UCLG	Self-evaluation	Yes	28	Cities willing to participate in the assessment survey have been selected (the tool developers made the methodology available online and invited cities to take part)
SCBC	The tool surveyors/auditors	Yes	28	No info

## 2 Experimental design, materials, and methods

The data presented in this dataset has been collected through detailed content analyses of documents related to the selected assessment schemes (i.e., manuals, scorecards, indices, rankings, working papers, and articles). To collect and analyze data reported in Table 1, Table 2, Table 3, Table 4, Table 5, Table 6, Table 7 and Fig. 1, Fig. 2, Fig. 3, Fig. 4, Fig. 5, Fig. 6, Fig. 7, Fig. 8, Fig. 9, Fig. 10, matrices were developed in Microsoft Excel to collect data related to different aspects of the schemes. Matrix rows and columns corresponded to selected tools and the explored aspects, respectively. After matrices were completed, whenever needed, simple statistical analyses were performed in Microsoft Excel to obtain the data reported in the remainder of the paper. The word cloud presented in Fig. 6 was created using the Microsoft Word App “Pro word Cloud”.

To create the matrix presented in Appendix I, first the most commonly-used themes and factors were identified. Next, a matrix was developed with rows corresponding to the themes and factors and columns corresponding to each of the selected assessment schemes. Next, documents and manuals related to each assessment scheme were explored to extract indicators related to each theme and factor. The process was started with the ASCIMER scheme. Indicators were incrementally added to the matrix while analyzing the other schemes. After completing the process for all selected schemes, simple statistical analyses were conducted using Microsoft Excel to identify the most persistently used indicators. For more details see Ref. [1]. A similar approach was taken for developing the matrix presented in Appendix II. The only difference is that in Appendix II matrix rows correspond to cities where selected assessment schemes have been implemented.
